# Compound‐specific amino acid ^15^N stable isotope probing of nitrogen assimilation by the soil microbial biomass using gas chromatography/combustion/isotope ratio mass spectrometry

**DOI:** 10.1002/rcm.7612

**Published:** 2016-07-31

**Authors:** A. F. Charteris, T. D. J. Knowles, K. Michaelides, R. P. Evershed

**Affiliations:** ^1^ Organic Geochemistry Unit, School of ChemistryUniversity of Bristol, Cantock's CloseBristolBS8 1TSUK; ^2^ School of Geographical SciencesUniversity of Bristol, University RoadBristolBS8 1SSUK

## Abstract

**Rationale:**

Organic nitrogen (N) greatly exceeds inorganic N in soils, but the complexity and heterogeneity of this important soil N pool make investigations into the fate of N‐containing additions and soil organic N cycling challenging. This paper details a novel approach to investigate the fate of applied N in soils, generating quantitative measures of microbial assimilation and of newly synthesized soil protein.

**Methods:**

Laboratory incubation experiments applying ^15^N‐ammonium, ^15^N‐nitrate and ^15^N‐glutamate were carried out and the high sensitivity and selectivity of gas chromatography/combustion/isotope ratio mass spectrometry (GC/C/IRMS) exploited for compound‐specific ^15^N stable isotope probing (^15^N‐SIP) of extracted incubation soil amino acids (AAs; as *N*‐acetyl, *O*‐isopropyl derivatives). We then describe the interpretation of these data to obtain a measure of the assimilation of the applied ^15^N‐labelled substrate by the soil microbial biomass and an estimate of newly synthesised soil protein.

**Results:**

The cycling of agriculturally relevant N additions is undetectable via bulk soil N content and *δ* ^15^N values and AA concentrations. The assimilation pathways of the three substrates were revealed via patterns in AA *δ* ^15^N values with time, reflecting known biosynthetic pathways (e.g. ammonium uptake occurs first via glutamate) and these data were used to expose differences in the rates and fluxes of the applied N substrates into the soil protein pool (glutamate > ammonium > nitrate).

**Conclusions:**

Our compound‐specific ^15^N‐SIP approach using GC/C/IRMS offers a number of insights, inaccessible via existing techniques, into the fate of applied ^15^N in soils and is potentially widely applicable to the study of N cycling in any soil, or indeed, in any complex ecosystem. © 2016 The Authors. *Rapid Communications in Mass Spectrometry* Published by John Wiley & Sons Ltd.

Organic nitrogen (N) concentrations far exceed those of inorganic N in most soils and, despite much investigation, the composition and cycling of this complex pool of soil organic matter (SOM) remain poorly understood (Fig. 1).^1^, ^2^, ^3^, ^4^, ^5^ A particular problem has been resolving more resistant soil organic N from that actively cycling through the soil system; an important consideration in soil N cycling studies, especially those focusing on nutrient supply. Studies monitoring the concentrations of added substrates and potential products (e.g.^4^) are useful because the concentrations of actively cycled components will fluctuate, providing indications of 'reactivity' in the soil. However, such approaches do not allow elucidation of the pathways of N transformation and are rather a blunt tool for interrogating soil‐based transformations critical to the global N cycle.

**Figure 1:**
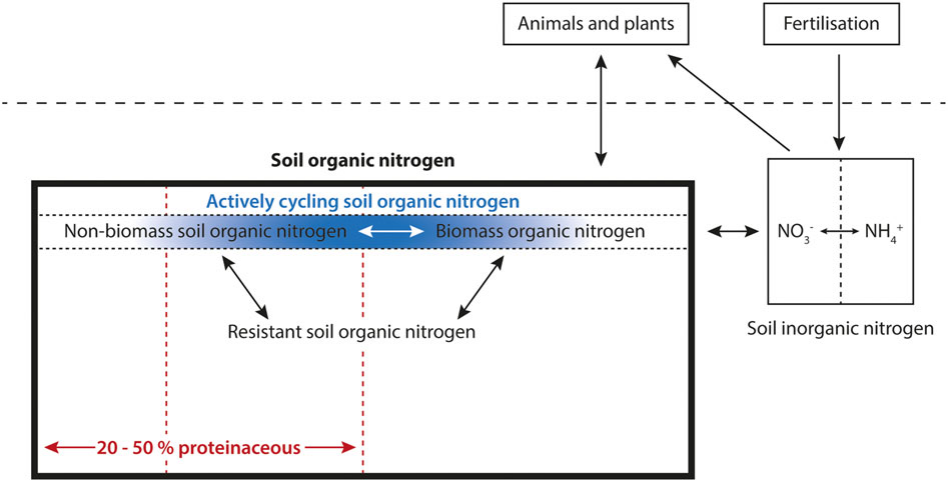
Conceptual diagram of the soil N cycle emphasising the relative contributions of organic and inorganic N to soil N (ca 90:10%) and the actively cycling soil organic N pool which is currently difficult to assess, but which may be estimated via the approach discussed in this paper. Inputs of the type applied in this study correspond to 'Fertilisation' and 'Animals and plants'.

The use of ^15^N‐labelled substrates as stable isotope tracers has contributed much to our understanding of N cycling in the soil system (e.g.^6^); however, the complexity and heterogeneity of soil organic N have prevented interrogation of the *biomolecular* fate of applied N in any detail. As a result, a considerable proportion of previous work has either assumed that since the majority of soil N is organic, all of the ^15^N retained in the soil is organic N (e.g.^7^), or has derived estimates for the N isotopic composition of organic N by extracting/subtracting ^15^N‐labelled inorganic compounds from bulk soils/values (e.g.^8^, ^9^). A shortcoming of both of these methods is that they only provide an estimate of the bulk N isotopic composition of what is an extremely complex and non‐uniformly ^15^N‐labelled organic N pool. Critically, in these methods the ^15^N substrates/amendments mostly serve as a physical tracer rather than a true biochemical tracer. A more refined approach has been to use microbial biomass N extraction^10^ and subsequent isotopic analysis to determine the N isotopic composition of biomass N, representing the fraction of ^15^N assimilated by microorganisms or the ^15^N cycling through the 'living', 'active' or 'available' portion of soil organic N.^11^, ^12^, ^13^ However, this extraction method can only generate estimates of bulk soil microbial biomass N.^14^, ^15^, ^16^, ^17^

A more recently developed technique that interrogates the active microbial community in more detail is ^15^N stable isotope probing (^15^N‐SIP) of nucleic acids (deoxyribonucleic acid (DNA) or ribonucleic acid (RNA)).^18^, ^19^, ^20^, ^21^ This culture‐independent method employs isopycnic centrifugation to separate out ^15^N‐enriched nucleic acids, which, following fingerprinting, may enable the taxonomic identity of actively assimilating microorganisms to be established.^18^, ^19^, ^20^ The technique is limited by: the high final nucleic acid ^15^N enrichments required (>50%); the potential for cross feeding and trophic cascades under highly ^15^N‐labelled substrates and longer incubation times; and the complexity of matching terminal restriction fragments (TRFs; which may be shared by multiple organisms) with sequences from clone libraries.^18^, ^19^, ^20^, ^22^, ^23^ In addition, although potentially valuable in identifying active microorganisms, nucleic acid ^15^N‐SIP does not afford insights into the N cycling of the soil community as a whole and the fate of applied ^15^N.

A technique which can offer this capability is compound‐specific ^15^N‐SIP of soil organic N using mass spectrometry. Many studies have used conventional gas chromatography/mass spectrometry (GC/MS) in compound‐specific ^15^N‐SIP studies (e.g. for ^15^N‐enriched amino sugars;^24^ for microbial amino acid (AA) utilisation;^25^ investigating barley leaf proteins with high turnover rates^26^), but this approach can generally only be used precisely (±0.01 atom %) with highly ^15^N‐enriched compounds where the ^15^N enrichment is easily detectable above natural background values.^27^, ^28^, ^29^ Only a handful of workers, however, have exploited the far higher potential precision (0.5–2.0 ‰; 0.0002–0.0008 atom %)^28^, ^29^, ^30^ of gas chromatography/combustion/isotope ratio mass spectrometry (GC/C/IRMS) in ^15^N‐SIP studies. Almost half of these relate to mammalian physiology (e.g.^31^, ^32^, ^33^) and the remainder consist of a few reports in several different research areas: N uptake in aquatic systems (e.g.^34^, ^35^, ^36^^]^); plant N uptake (e.g.^37^, ^38^); plant‐microbe associations (e.g.^39^); microbial cultures;^40^ and soil N partitioning (e.g.^41^, ^42^, ^43^).

The latter studies have begun to hint at the potential of ^15^N‐SIP using GC/C/IRMS as an extremely powerful tool for tracing the fate of ^15^N in soils. The critical advantage is that this technique offers the sensitivity required to follow ^15^N substrates applied at environmentally relevant concentrations and appropriately low enrichments, through a variety of ecosystems and into a range of N‐containing products. Given this, it is surprising that this approach remains largely unexploited and, hence, the full range of applications and insights is yet to be realised. This may be partly due to the challenges associated with compound‐specific ^15^N analyses via GC/C/IRMS, as compared with those of carbon‐13 (^13^C; N is generally much less abundant than C in organic molecules; two N atoms are required to produce each N_2_ molecule for analysis; additional reduction chemistry is required to successfully convert a N‐containing molecule into N_2_ for analysis; the ionisation efficiency of N_2_ is only 70% that of carbon dioxide (CO_2_); small leaks can be detrimental due to the high abundance of N_2_ in air; and there is potential for interfering ionic species, such as [CO]^+^, at *m*/*z* 28, 29 and 30),^44^, ^45^ which can make the technique somewhat temperamental, but may also be due to a lack of awareness regarding the potential of compound‐specific ^15^N‐SIP using GC/C/IRMS to investigate soil N cycling.

Herein we describe the advantages of compound‐specific AA ^15^N‐SIP using GC/C/IRMS to investigate the fate of N in soils and obtain a measure of the assimilation of an applied ^15^N‐labelled substrate by the soil microbial biomass. We demonstrate the utility of the approach for the study of any N‐containing soil amendment via the results of laboratory incubations applying inorganic (^15^N‐ammonium; [^15^NH_4_]^+^, ^15^N‐nitrate; [^15^NO_3_]^–^) and organic (^15^N‐glutamate; ^15^N‐Glu) substrates. We derive quantitative estimates of newly synthesised soil protein, which is representative of the functioning of the soil microbial biomass and biomass protein production using the applied substrate. The high selectivity and sensitivity of GC/C/IRMS^29^, ^46^, ^47^, ^48^ enable the use of environmentally relevant ^15^N‐tracer doses that minimise perturbations to native soil conditions and ensure the highly 'diluted' metabolic products of the ^15^N tracer are readily detectable in the incubated soils. The power of the approach lies in the analysis of AAs as these are major 'building blocks' of all life, forming the proteins which regulate essential biochemical reactions. Proteinaceous matter (proteins, peptides and AAs) generally comprises 20–50% of total soil N and is ubiquitous in living organisms, so is a major 'organic product' of microbial activity/assimilation.^1^, ^2^, ^3^, ^49^ Since AAs represent major organic nitrogenous products in soil they provide a highly sensitive integrating tool across the many thousands of proteins present, revealing important general features of the dynamics and pathways of assimilation of N‐containing substrates into the organic N pool. Critically, the percentage of applied ^15^N detectable in the total hydrolysable AA pool offers a measure of (or 'proxy' for) the assimilation of applied ^15^N‐labelled substrate by the soil microbial biomass and an estimate of newly synthesised soil protein. We discuss the range of potential insights and highlight the wider applicability of the approach in the investigation of complex N cycling ecosystems.

## Experimental

### Incubations

Soil was sampled randomly from Rowden Moor experimental site (plot six) at North Wyke Research Station near Okehampton, Devon, UK; the same site used by Knowles *et al.* 41 The soil is classified as a clayey non‐calcareous Pelostagnogley of the Hallsworth series (British Classification), a Stagni‐vertic cambisol under the Food and Agriculture Organisation of the United Nations (FAO) scheme or a Typic haplaquept by the United States Department of Agriculture (USDA).^50^ Sampled soils were homogenised, air‐dried to allow sieving (2 mm) and then adjusted to 50% water holding capacity (WHC) by the addition of double distilled water (DDW). Incubations were carried out in small glass tubes (10 cm height × 2 cm diameter) containing 10 g soil under aerobic conditions and maintained by weight at 50% WHC. After a 4‐day pre‐incubation period to allow for equilibration to the new conditions, the microcosms received treatments of either ^15^N‐labelled ammonium chloride (^15^NH_4_Cl, 10 atom %, 400 μg in 200 μL DDW; Sigma‐Aldrich, St. Louis, MO, USA), ^15^N‐labelled potassium nitrate (K^15^NO_3_, 10 atom %, 400 μg in 200 μL DDW; Sigma‐Aldrich), ^15^N‐labelled glutamic acid (^15^N‐Glu, 98 atom %, 2 mg in 200 μL 0.1 M hydrochloric acid; HCl; ^15^N‐Glu from Spectra Stable Isotopes, Columbia, MD, USA and HCl was reagent grade from Fisher Scientific, Loughborough, UK) or for the control samples, DDW (200 μL). Substrates were introduced by injection and the needle was drawn up through the soil as the plunger was depressed in order to achieve an optimal distribution. The incubation experiments were halted by immersion in liquid nitrogen (N_2_) after periods of 1.5, 3, 6 and 12 h and 1, 2, 4, 8, 16 and 32 days in the dark and stored at 20 °C until freeze‐drying. All incubations were carried out in triplicate so there were three tubes for each time point of each treatment (see also Knowles *et al.* 41). The 10 atom % ^15^N enrichment of ^15^[NH_4_]^+^ and ^15^[NO_3_]^–^ was chosen based on research highlighting changes in ^15^N discrimination and isotopic fractionation in biological mechanisms at very high enrichments.^51^, ^52^ ^15^N enrichments of 10 atom % were considered low enough for these effects to be negligible. The ^15^N‐Glu incubation experiments were carried out earlier; hence the high ^15^N enrichment of the applied Glu.

### Extraction, isolation and derivatisation of hydrolysable AAs

Finely ground, freeze‐dried incubation soil samples (100 mg) were weighed into culture tubes and 100 μL of norleucine (Nle; 400 μg mL^–1^ in 0.1 M HCl; Sigma‐Aldrich) was added as an internal standard. Hydrolysis with 5 mL 6 M HCl was carried out at 100 °C for 24 h under an atmosphere of N_2_. Acid hydrolysis extracts both free and proteinaceous AAs as well as catalysing the breakdown of living microbial biomass.^49^ The relatively harsh conditions are necessary for the cleavage of peptide bonds between hydrophobic residues (e.g. isoleucine; Ile, leucine; Leu and valine; Val), but also result in the deamination of asparagines (Asn) to aspartate (Asp) and glutamine (Gln) to Glu and the complete destruction of cysteine (Cys) and tryptophan (Trp).^49^, ^53^ The technique may also partially destroy serine (Ser; ca 10% loss), threonine (Thr; ca 5% loss) and tyrosine (Tyr; loss depends on level of trace impurities in hydrolysis agent)^53^ and has the potential to hydrolyse AA chains from non‐proteinaceous sources, such as peptidoglycan, resulting in an overestimation of some AAs, mostly alanine (Ala), Glu, lysine (Lys) and glycine (Gly).^49^ The technique is, however, considered the most reliable method for determining the total protein content of soils^49^ and, as such, we equate total hydrolysable AA concentrations to the size of the soil protein pool. The hydrolysis is performed under N_2_ as the presence of oxygen (O_2_) can induce the thermal breakdown of hydroxyl‐ and sulfur‐containing AAs (e.g. methionine; Met, Ser, Thr and Tyr).^49^

Hydrolysates were collected by centrifugation, dried under a stream of N_2_ at 60 °C and stored at 20 °C under 1 mL 0.1 M HCl. AAs were isolated from hydrolysates by cation‐exchange column chromatography using acidified Dowex 50WX8 200–400 mesh ion‐exchange resin (Acros Organics, Morris Plains, NJ, USA).^54^ This was followed by conversion into their *N*‐acetyl, *O*‐isopropyl derivatives for analysis.^41^, ^55^ Derivatising agents were supplied by Sigma‐Aldrich (Steinheim, Germany): acetyl chloride was puriss. p.a. grade; trimethylamine had ≥99.50% purity; and acetic anhydride was ReagentPlus® grade. All solvents were of HPLC grade and were supplied by Rathburn Chemicals Ltd. (Walkerburn, UK). DDW was produced using a Bibby Aquatron still. Where not applied to living soil, DDW was extracted with dichloromethane prior to use in order to remove dissolved organic compounds.

### Instrumental analyses

Bulk soil percentage total N (% TN) and *δ* ^15^N analyses were carried out using a Eurovector elemental analyser (Milan, Italy) coupled to a Micromass Isoprime isotope ratio mass spectrometer (Stockport, UK) at the Lancaster node of the Natural Environment Research Council Life Sciences Mass Spectrometry Facility (NERC LSMSF; UK). Soil (ca 10 mg) was weighed into tin capsules, combusted and subsequently reduced over heated copper (Cu) wires in the elemental analyser before the resultant N_2_ was passed into the isotope ratio mass spectrometer for determination of % TN contents and *δ* ^15^N values.

A model 5890 Series II gas chromatograph (Hewlett Packard, Wilmington, DE, USA) fitted with a *VF‐23*ms column (60 m × 0.32 mm i.d., 0.15 μm phase thickness; Varian Inc., Palo Alto, CA, USA) and a flame ionisation detector (FID; Hewlett Packard) was used for quantification of individual AAs as their *N*‐acetyl, *O*‐isopropyl derivatives by comparison with the internal standard, Nle. The *N*‐acetyl, *O*‐isopropyl AAs were identified by their known elution order^55^ and by comparison with AA standards (TLC grade, 98%; Sigma‐Aldrich). The carrier gas was hydrogen (H_2_; The BOC Group plc, Guildford, UK), at a flow rate of 3 mL min^–1^. The temperature programme utilised was: 40 °C (1 min) to 120 °C at 15 °C min^–1^, then to 190 °C at 3 °C min^–1^ and finally to 260 °C (12 min) at 5 °C min^–1^. Data were acquired and analysed using Clarity chromatographic station for Windows by DataApex (Prague, Czech Republic).

**Figure 2:**
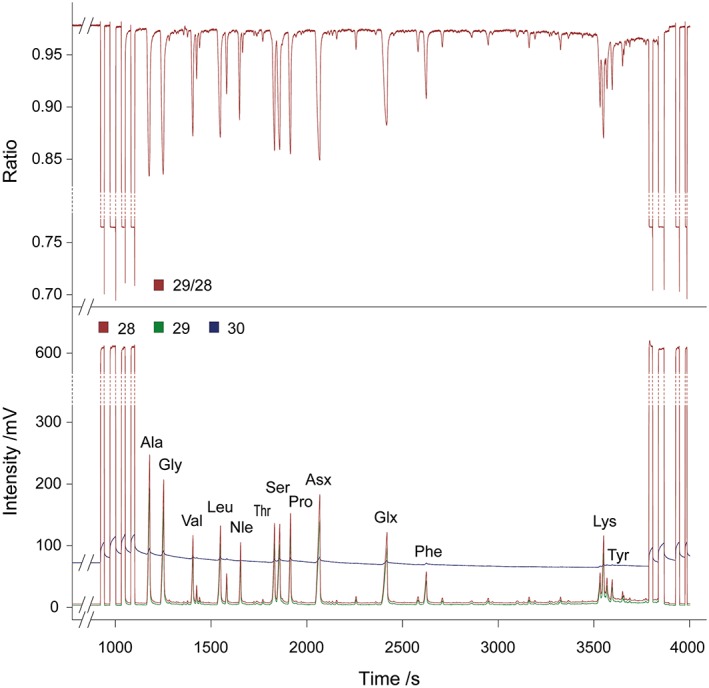
Typical GC/C/IRMS chromatogram of *N*‐acetyl, *O*‐isopropyl derivatised hydrolysable soil AAs showing the ion current signals recorded by GC/C/IRMS operating for N_2_ (*m*/*z* 28, 29 and 30) and the ratio of *m*/*z* 28 to *m*/*z* 29 which is used to generate ^15^N/^14^N isotope ratios.

The *δ* ^15^N values of individual AAs as their *N*‐acetyl, *O*‐isopropyl derivatives were determined using a ThermoFinnigan Trace 2000 gas chromatograph coupled with a ThermoFinnigan DeltaPlus XP isotope ratio mass spectrometer via a ThermoFinnigan Combustion III Interface (Thermo Electron Corporation, Waltham, MA, USA). Samples were introduced using a GC Pal autosampler (CTC Analytics, Zwingen, Switzerland) and via a programmable temperature vaporisation (PTV) inlet (Thermo Electron Corporation). The carrier gas was helium (He; The BOC Group plc) at a flow rate of 1.4 mL min^–1^ and the gas chromatograph was fitted with a DB‐35 column (30 m × 0.32 mm i.d. × 0.5 μm stationary phase thickness; Agilent Technologies, Santa Clara, USA). The temperature programme utilised was: 40 °C (5 min) to 120 °C at 15 °C min^–1^, to 180 °C at 3 °C min^–1^, then to 210 °C at 1.5 °C min^–1^ and finally to 270 °C at 5 °C min^–1^. The oxidation reactor was composed of Cu, nickel (Ni) and platinum (Pt) wires (high purity from OEA Laboratories Ltd, Callington, UK) and maintained at 980 °C and the reduction reactor was composed of Cu wires and maintained at 650 °C. AA *δ* ^15^N values were determined relative to that of a monitoring gas of known (previously determined using in‐house AA standards) N isotopic composition introduced directly into the ion source via an open split in four pulses at the beginning and end of each run. The *δ* ^15^N values of the in‐house AA standards were determined off‐line by elemental analysis/isotope ratio mass spectrometry (EA/IRMS) by Thermo Fisher Scientific (Bremen, Germany) and by the NERC Centre for Ecology & Hydrology (CEH; Merlewood/Lancaster, UK) using primary reference materials (NIST 8547 IAEA‐N‐1 ammonium sulfate; *δ* ^15^N +0.4 ‰). In order to adhere to the identical treatment principle and ensure the GC/C/IRMS system was functioning properly, each sample was bracketed by the in‐house AA standard mixture of known *δ* ^15^N values and sample AA *δ* ^15^N values accepted only when at least 75% of the AAs in the standard mixture run either side of the sample were within ±1 ‰ and the others were within ±1.5 ‰, and when this was also true on average over the course of the run. Data were acquired and analysed using Isodat NT 3.0 (Thermo Electron Corporation). Figure 2 shows a typical sample chromatogram including the ion current signals for each *m*/*z* value recorded.

## Calculations

If the total hydrolysable AA pool is taken to be representative of the soil protein pool, then any ^15^N enrichment (*E*) in hydrolysable AAs can be summed to represent newly synthesised soil protein in the soil at that time:

(1) Newly synthesised soil protein≈∑Ein hydrolysableAAs

The *E* of an AA may be expressed as the number of moles of ^15^N derived from the applied substrate that are present in that AA in the soil:

(2) $$E=n_{N}\times \mathrm{AFE}$$

where *n*_N_ is the number of moles of N in the AA (i.e. if the molecular structure of the AA contains only one N atom, *n*_N_ is the same as the number of moles of the AA in the soil, but twice this if the AA structure consists of two N atoms and so on) and AFE is the atom fraction excess of the AA after incubation compared with the control:

(3) $$\mathrm{AFE}={\mathrm{AF}}_{\text{Sample}}-{\mathrm{AF}}_{\text{Control}}$$

AF is the atom fraction of ^15^N in the AA, i.e.:

(4) NumberN15atoms(N14+N)atoms15

This can be calculated from the AA's *δ* ^15^N value as in Knowles *et al*.:^41^

(5) AF=RStdδN15/1000+11+δN15/1000+1

where *R*_Std_ is the ^15^N/^14^N ratio of AIR, the international isotopic standard for N. The data may also be expressed in terms of the percentage of the applied ^15^N incorporated into each AA, as in Knowles *et al*.:^41^

(6) $$\%\;\text{Incorporation}=\left(\frac{E}{N}\right)\times 100$$

where *N* is the number of moles of excess ^15^N applied (above natural abundance). Percentage incorporations reflect both the concentration and ^15^N enrichment (*δ* ^15^N value) of the AA (i.e. how much was incorporated if the AA at *x* concentration was ^15^N‐enriched by *x* ‰) and the percentage of applied ^15^N incorporated into newly synthesised soil protein is determined by summing these results for individual AAs. Note that these percentage incorporation data will be affected by the conservation of applied ^15^N in the system; thus, if ^15^N is lost from the system (e.g. over time), AA percentage incorporations may become skewed as there is less ^15^N available for incorporation than expected. The incubation experiment design described aims to limit any ^15^N losses from the system in order to obviate this issue, but it is equally possible to calculate percentage incorporations at time, *t*, based on the moles of applied ^15^N retained (*N*_R_; above natural abundance/control soil values) in the system at time, *t* (Eqn. (7)), if bulk recovery of the applied ^15^N is low or decreases with time.

(7) $$N_{R}=\mathrm{AFE}\left(\frac{\%\mathrm{TN}}{1400}\right)\;$$

where AFE is the atom fraction excess of ^15^N in the bulk soil (calculated from bulk and control soil *δ* ^15^N values using Eqns. (3) and (5)) and % TN is the percentage total N content of the soil. Percentage retention of applied ^15^N was calculated as follows:

(8) %RetentionN=NRN×10015

It can also be argued, however, that loss from the system is just another process competing against AA biosynthesis for N, so should not be discounted in this way. All of these approaches are valid and the most appropriate one will depend on the specifics of the experimental design and desired outcomes.

## Results and Discussion

Compound‐specific AA ^15^N‐SIP using GC/C/IRMS has the potential to provide hitherto unattainable insights into soil N cycling (Fig. 1) from any (inorganic or organic) N‐containing substrate (≡ amendments). The utility of the method is discussed in terms of: (i) limitations of bulk N and AA concentrations to detect appropriate N additions/cycling in soils; (ii) pathways of assimilation of different N‐containing substrates; (iii) revealing differences in rates and fluxes of N between applied substrates; and (iv) interpretations of ^15^N‐SIP determinations in relation to complex N dynamics in soils.

### Limitations of bulk N and AA concentrations to detect appropriate N additions/cycling in soils

The addition of an agriculturally relevant, but sufficiently low, N concentration to prevent alteration of the soil's N status (and thereby limit perturbation) almost by definition results in no notable changes in the % TN of the soil over the course of the experiment. Tables 1, 2 and 3 confirm this – there is no observable trend in the % TN of the incubation microcosms and the standard errors of the means (SEs) of the % TN contents for all incubation microcosms are small. Thus, the application of ^15^N‐labelled amendments is clearly valuable in allowing added N to be differentiated from native soil N. However, following addition of all three substrates, bulk soil *δ* ^15^N values, following the initial rise, remained relatively constant throughout the rest of the incubation experiment (i.e. overall percentage retentions of ^15^N in the system were high and close to 100%; Table 4). The elevated *δ* ^15^N values compared to *t* = 0 values confirm the continued presence of the ^15^N tracer in the soil, but no insights can be gained about the form or internal processing of the amendments within the soil, i.e. is the ^15^N still present as ^15^[NH_4_]^+^, ^15^[NO_3_]^–^ or ^15^N‐Glu or has it been assimilated by the soil microbial biomass?

**Table 1:** Soil % TN and composition and concentrations of soil hydrolysable AAs for the ^15^[NH_4_]^+^‐SIP experiment

		Time /days												
		0	0.0625	0.125	0.25	0.5	1	2	4	8	16	32	Mean	SE
Mean concentration /mg g^−1^	% TN	0.63	0.60	0.62	0.62	0.62	0.56	0.55	0.67	0.69	0.64	0.69	0.63	0.0081
	Ala	2.38	1.92	1.79	1.84	1.91	1.87	1.93	1.90	2.02	2.11	2.09	2.03	0.0453
	Asx	1.61	2.21	1.80	1.82	2.01	1.94	2.10	2.00	2.11	2.08	2.03	1.95	0.0512
	Glx	1.59	2.02	1.81	1.82	1.88	1.81	1.89	1.83	1.92	1.89	1.88	1.83	0.0440
	Gly	1.83	1.26	1.14	1.41	1.34	1.33	1.52	1.37	1.49	1.49	1.44	1.46	0.0435
	Hyp	0.12	0.12	0.11	0.12	0.12	0.11	0.12	0.11	0.13	0.13	0.13	0.12	0.0024
	Ile	0.38	0.61	0.54	0.43	0.46	0.52	0.55	0.45	0.48	0.48	0.47	0.48	0.014
	Leu	1.02	1.16	1.15	1.05	1.07	1.05	1.09	1.03	1.09	1.13	1.12	1.09	0.0119
	Lys	0.48	0.41	0.27	0.27	0.25	0.34	0.48	0.40	0.51	0.59	0.55	0.42	0.029
	Met	0.07	0.12	0.12	0.09	0.13	0.10	0.08	0.13	0.12	0.11	0.13	0.1	0.005
	Phe	0.48	0.55	0.58	0.45	0.46	0.46	0.48	0.54	0.60	0.59	0.64	0.53	0.014
	Pro	1.23	1.14	1.10	1.19	1.14	1.09	1.14	1.07	1.14	1.18	1.20	1.16	0.0197
	Ser	0.89	1.02	0.85	0.84	0.94	0.92	1.04	0.95	1.07	1.12	1.05	0.97	0.023
	Thr	0.73	1.07	0.89	0.87	0.90	0.86	0.96	0.92	0.99	1.01	0.93	0.90	0.025
	Tyr	0.22	0.29	0.29	0.23	0.24	0.24	0.26	0.30	0.36	0.35	0.35	0.28	0.0099
	Val	0.75	0.95	0.80	0.76	0.72	0.83	0.90	0.78	0.82	0.83	0.78	0.81	0.022
	THAA N	13.8	14.9	13.2	13.2	13.6	13.5	14.5	13.8	14.8	15.1	14.8	14.1	0.197
	% THAA N of TN	28.8	30.5	26.4	26.5	27.5	30.3	33.5	25.9	27.2	29.9	26.8	28.5	0.555

THAA N; total hydrolysable amino acid nitrogen.

**Table 2:** Soil % TN and composition and concentrations of soil hydrolysable AAs for the ^15^[NO_3_]^−^‐SIP experiment

		Time /days												
		0	0.0625	0.125	0.25	0.5	1	2	4	8	16	32	Mean	SE
Mean concentration /mg g^−1^	% TN	0.63	0.67	0.65	0.67	0.66	0.65	0.67	0.66	0.66	0.68	0.69	0.66	0.0039
	Ala	2.38	1.86	1.93	1.97	1.93	2.11	1.97	2.18	1.81	1.85	2.32	2.06	0.0558
	Asx	1.61	1.92	1.99	2.02	2.01	1.83	2.19	1.82	1.55	1.56	1.15	1.77	0.0660
	Glx	1.59	1.78	1.85	1.73	1.81	1.83	1.92	1.80	1.51	1.57	1.13	1.68	0.0551
	Gly	1.83	1.27	1.39	1.38	1.36	1.53	1.42	1.42	1.20	1.47	1.82	1.49	0.0503
	Hyp	0.12	0.12	0.12	0.12	0.12	0.14	0.12	0.13	0.11	0.10	0.12	0.12	0.0028
	Ile	0.38	0.49	0.49	0.39	0.35	0.25	0.34	0.39	0.34	0.26	0.37	0.37	0.015
	Leu	1.02	1.07	1.11	1.02	0.97	0.95	0.95	1.07	0.85	0.91	0.87	0.98	0.017
	Lys	0.48	0.34	0.41	0.57	0.46	0.39	0.40	0.46	0.53	0.39	0.60	0.44	0.025
	Met	0.07	0.13	0.12	0.12	0.10	0.09	0.10	0.07	0.06	0.08	0.06	0.09	0.004
	Phe	0.48	0.58	0.59	0.59	0.54	0.56	0.43	0.51	0.37	0.49	0.41	0.50	0.015
	Pro	1.23	1.11	1.14	1.08	1.10	1.23	1.12	1.22	1.01	0.97	1.23	1.14	0.0261
	Ser	0.89	0.89	0.95	1.12	1.05	0.90	1.00	0.98	0.87	0.81	0.85	0.93	0.026
	Thr	0.73	0.82	0.90	0.97	0.88	0.68	0.87	0.86	0.77	0.67	0.65	0.80	0.027
	Tyr	0.22	0.34	0.31	0.37	0.34	0.34	0.23	0.26	0.19	0.25	0.21	0.27	0.012
	Val	0.75	0.72	0.77	0.73	0.63	0.44	0.63	0.76	0.67	0.52	0.62	0.67	0.026
	THAA N	13.8	13.4	14.1	14.2	13.6	13.3	13.7	13.9	11.7	11.9	12.4	13.3	0.245
	% THAA N of TN	28.8	25.0	27.1	26.6	26.3	26.1	25.8	27.0	22.6	22.6	24.2	25.9	0.523

THAA N; total hydrolysable amino acid nitrogen.

**Table 3:** Soil % TN and composition and concentrations of soil hydrolysable AAs for the ^15^N‐Glu‐SIP experiment

		Time / days											
		0	0.125	0.25	0.5	1	2	4	8	16	32	Mean	SE
Mean concentration /mg g^−1^	% TN	0.76	0.76	0.78	0.77	0.79	0.77	0.76	0.77	0.78	0.77	0.77	0.0033
	Ala	5.41	7.87	7.35	7.44	5.73	4.12	4.38	5.46	3.45	4.47	5.58	0.387
	Asx	3.60	4.22	3.75	3.17	3.56	3.11	3.61	3.26	2.21	2.38	3.26	0.189
	Glx	2.93	3.88	3.60	3.08	2.83	2.54	3.01	2.97	1.91	2.04	2.88	0.168
	Gly	4.71	6.38	6.02	6.17	4.82	3.67	3.54	5.19	3.19	3.76	4.75	0.297
	Ile	1.25	1.44	1.40	1.32	1.15	0.94	0.97	2.34	1.01	0.72	1.3	0.12
	Leu	0.69	0.97	0.90	0.78	0.70	0.53	0.84	0.60	0.33	0.48	0.68	0.047
	Lys	0.64	0.21	0.14	0.32	0.15	0.92	0.46	1.30	0.98	0.28	0.53	0.078
	Met	0.17	0.23	0.23	0.20	0.17	0.17	0.24	0.14	0.07	0.10	0.2	0.01
	Phe	0.41	0.40	0.41	0.40	0.36	0.35	0.36	0.49	0.28	0.26	0.37	0.020
	Pro	2.51	3.57	3.79	3.66	2.69	1.81	2.23	3.01	1.64	2.26	2.73	0.197
	Ser	2.75	3.69	3.33	3.03	2.53	2.17	2.50	2.59	1.79	1.77	2.61	0.159
	Thr	2.28	2.24	1.91	1.68	1.95	1.38	1.88	1.81	1.17	1.08	1.70	0.106
	Val	1.29	1.39	1.39	1.05	1.07	0.51	1.00	0.75	0.46	0.48	0.91	0.079
	THAA N	28.6	36.5	34.2	32.3	27.7	22.2	25.0	29.9	18.5	20.1	27.4	1.46
	% THAA N of TN	50.6	65.6	60.0	57.7	47.2	39.1	43.6	52.9	32.5	35.9	48.4	2.71

THAA N; total hydrolysable amino acid nitrogen. (Note that soil used in the ^15^N‐Glu‐SIP incubation experiment was sampled at a different time from soil for the ^15^[NH_4_]^+^‐ and ^15^[NO_3_]^−^‐SIP incubation experiments and this has resulted in the higher concentrations of hydrolysable AAs observed throughout.)

**Table 4:** Bulk soil *δ* ^15^N values for the ^15^[NH_4_]^+^, ^15^[NO_3_]^−^ and ^15^N‐Glu incubation experiments

	*t* = 0		*t* = 3 h		Overall incubation mean			
	Mean *δ* ^15^N value	SE	Mean *δ* ^15^N value	SE	Mean *δ* ^15^N value	SE	% Retention ^15^N	SE
^15^NH_4_^+^	4.47	0.0432	87.3	4.03	85.4	1.25	109	2.07
^15^NO_3_^−^	4.47	0.0432	35.8	1.69	36.8	1.09	88	3.1
^15^N‐Glu	7.16	0.773	1050	67.0	1070	14.0	100	1.32

As essential biomolecules, proteinaceous AAs are likely products of amendment assimilation; however, the concentrations of individual AAs show little change over the course of the incubation and there is no observable increase in concentration with incubation duration, as might be expected from the synthesis of new AAs using the applied [NH_4_]^+^, [NO_3_]^–^ or Glu (Tables 1, 2 and 3). This could imply that the supplied [NH_4_]^+^, [NO_3_]^–^ or N‐Glu has not been used in the synthesis of AAs, but it could also be that the concentration of [NH_4_]^+^, [NO_3_]^–^ or N‐Glu added has not stimulated protein biosynthesis above that present prior to the additions. Accordingly, the total hydrolysable AA N content of the soil is on average 28.5% (SE: 0.555), 25.9% (SE: 0.523) and 48.4% (SE: 2.71) of total soil N throughout the ^15^[NH_4_]^+^, ^15^[NO_3_]^–^ or ^15^N‐Glu experiments, respectively (Tables 1, 2 and 3). These concentrations fall within the range (20–50%) generally reported for total hydrolysable soil AAs^1^, ^2^, ^3^, ^49^ and are equated to the concentration of the soil protein pool.^49^ We note that acid hydrolysis does not extract all proteinaceous AAs and extracts some non‐proteinaceous AAs;^3^, ^49^ however, the fraction of soil AAs recovered is constant. These results emphasise the need to undertake compound‐specific N isotope analysis using GC/C/IRMS of the newly biosynthesised AAs (mostly new protein) to gain detailed insights into the dynamics of the assimilation of a ^15^N‐containing amendment into the soil organic N pool.

### Pathways of assimilation of different N‐containing substrates

**Figure 3:**
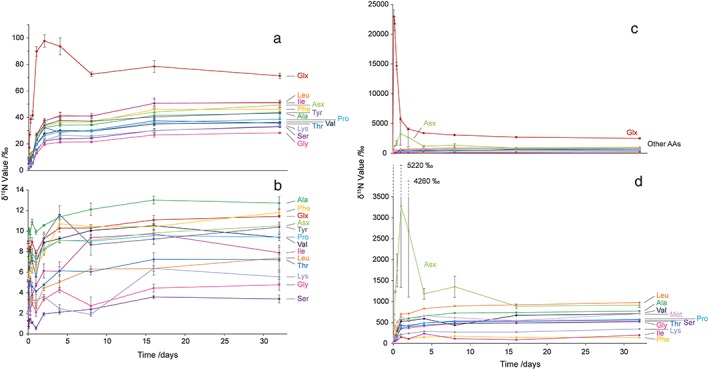
*δ* ^15^N values of individual AAs over the course of a 32‐day incubation experiment: (a) ^15^[NH_4_]^+^ incubation, (b) ^15^[NO_3_]^–^ incubation, (c) ^15^N‐Glu incubation, including the applied ^15^N‐Glu and (d) ^15^N‐Glu incubation, excluding the applied ^15^N‐Glu. Error bars are ± SE (n = 3).

The use of GC/C/IRMS allows precise (0.5–2.0 ‰; 0.0002–0.0008 atom %) determination of the *δ* ^15^N values of individual hydrolysable soil AAs.^28^, ^29^, ^30^ Figure 3 shows the trends in hydrolysable AA *δ* ^15^N values over the course of the incubation experiments applying ^15^[NH_4_]^+^, ^15^[NO_3_]^–^ or ^15^N‐Glu. The advantages of the compound‐specific approach are immediately apparent, with readily detectable changes being seen in the *δ* ^15^N values of all AAs in all experiments (Fig. 3). It should be noted that this is the first time the assimilation of [NH_4_]^+^ and [NO_3_]^–^ by the soil microbial biomass has been measured in this way and the results clearly emphasise the importance of investigating N cycling from different N‐containing soil amendments.

Incorporation of ^15^[NH_4_]^+^ (Fig. 3(a)) occurs in two phases for all AAs except Glx – fast over the first 2 to 4 days, then more slowly for the remainder of the experiment. Assimilation into Glx occurs more quickly over the first 2 days than into any other AA and the degree of ^15^N enrichment is 2‐ to 5‐fold greater, before declining during the rest of the experiment. These differences in the patterns of ^15^N incorporation relate to the fundamental biosynthetic pathways most microorganisms use for the assimilation of [NH_4_]^+^, i.e. via the reductive amination of α‐ketoglutarate to L‐Glu catalysed by glutamate dehydrogenase (GDH) or via the glutamine synthetase (GS), glutamate synthase (glutamine oxoglutarate aminotransferase; GOGAT) pathway.^56^, ^57^, ^58^ Glu is of central importance to the biosynthesis of new proteins as other AAs are synthesised from Glu, using it as a substrate for the amination of appropriate α‐ketoacid C skeletons. The fast rise in the *δ* ^15^N values of Glu reflects the initial incorporation of ^15^[NH_4_]^+^, with the subsequent decline after 2 days reflecting redistribution of the ^15^N into newly synthesised AAs, hence their *δ* ^15^N values rise.

The results for the ^15^[NO_3_]^–^ experiment are extremely interesting, further emphasising the importance of this approach (Fig. 3(b)). Broadly, most AAs initially, and somewhat surprisingly, show lower *δ* ^15^N values before rising slightly over the rest of the incubation. The initial dip indicates that at the start of the incubation AAs are ^15^N‐depleted compared with at *t* = 0. The reason for this is unknown, but one possibility is that contact with initial high [NO_3_]^–^ concentrations causes cell lysis providing non‐proteinaceous substrates with low *δ* ^15^N values for AA biosynthesis. The subsequent smaller and more irregular rise in *δ* ^15^N values for all AAs compared to the ^15^[NH_4_]^+^ experiment is likely because [NO_3_]^–^ requires reduction prior to incorporation into AAs.^59^

Comparison with the ^15^N‐Glu incubation carried out by Knowles *et al.* 41 (data represented for this discussion in Figs. 3(c) and 3(d)) exposes a more complex situation as the applied substrate is itself an AA in the total hydrolysable AA pool. As might be expected, the *δ* ^15^N value of Glu falls with time, whilst those of the other AAs rise (Figs. 3(c) and 3(d)). Interestingly, a comparable pattern to that of ^15^[NH_4_]^+^ incorporation emerges for the transfer of ^15^N‐Glu into other hydrolysable AAs, but in this case all non‐substrate AAs, except Asx, exhibit two‐phase incorporation. As Knowles *et al.* 41 concluded, this is again likely due to the fundamental biosynthetic pathways that operate in most microorganisms; Asp is produced by the transamination of oxaloacetate using an amino group from Glu,^60^ the remaining C‐skeleton of which is α‐ketoglutarate, which is used in the tricarboxylic acid (TCA) cycle, an essential metabolic process that generates energy in aerobic respiration. Decarboxylation of α‐ketoglutarate as part of the cycle then generates another molecule of oxaloacetate. Interpreting the rate data alongside this known biochemistry, Knowles *et al.* 41 concluded that the patterns of isotope incorporation are consistent with Asp being the AA closest in biosynthetic proximity to Glu.

### Revealing differences in rates and fluxes of N between applied substrates

**Figure 4:**
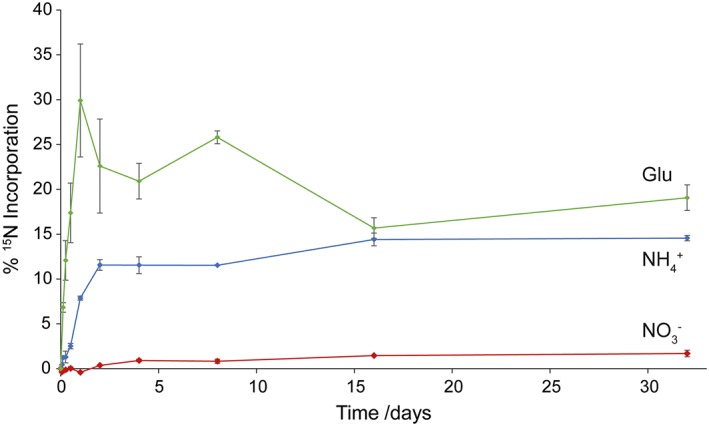
Percentage of applied ^15^[NH_4_]^+^, ^15^[NO_3_]^–^ and ^15^N‐Glu incorporated into the total hydrolysable AA pool or soil protein pool. Error bars are ± SE (n = 3). Calculations for ^15^[NH_4_]^+^ and ^15^[NO_3_]^–^ are straightforward summations of the percentage of the applied ^15^N incorporated into each AA, while results for ^15^N‐Glu incubation were, in this case, calculated excluding the ^15^N residing in Glu as a relatively high level of enrichment remains at the apparent equilibrium compared with the enrichment of the other AAs (Fig. 2(c)) indicating considerable intact use of the applied ^15^N in preference to *de novo* AA biosynthesis.

Quantifying the fate of N‐containing substrates (inorganic or organic) in different soils is essential to understanding the N cycle in natural or semi‐natural ecosystems but is especially important in agricultural systems where managing fertiliser applications has ecological and economic relevance. The new insights gained into N cycling through this novel approach offer potential to enhance fundamental understanding in this area. Using Eqns. (3)–(6), increases in AA *δ* ^15^N values can be used to determine the percentage of the applied ^15^N incorporated into each AA and, by summation, the percentage incorporated into the total hydrolysable AA or soil protein pool and cycling through the 'living', 'active' or 'available' portion of soil organic N at that time (Fig. 4). These calculations are straightforward where the applied substrate is not a hydrolysable AA (e.g. ^15^[NH_4_]^+^ and ^15^[NO_3_]^–^) as any ^15^N enrichment in the hydrolysable AA pool must be derived from the applied substrate via microbial processing during the experiment. The assessment is more complicated however when the applied substrate is a hydrolysable AA (e.g. ^15^N‐Glu) as this must be accounted for in the analytical approach^41^ and calculations (Fig. 4).

The use of several different treatments applied separately to the same soil allows comparison of their relative 'availabilities' to the soil microbial biomass – in the case of [NH_4_]^+^, [NO_3_]^–^ and Glu here, clear differences in the assimilation of these substrates into newly synthesised hydrolysable soil AAs are revealed. Alternatively, the technique can also be used to compare the fate of particular N amendments in different soils to provide hitherto unattainable estimates of the relative 'activity' of the microbial biomass of the soils under selected incubation conditions. In both cases, a measure of newly synthesised protein can be obtained by summing the ^15^N enrichments of all the AAs at each time point for each treatment (Eqns. (1) and (2)) to give the moles of ^15^N in the soil protein pool at that time. Note, however, that new protein will also be biosynthesised from non‐labelled sources during the experiment, e.g. following cell lysis or concomitant organic matter mineralisation.

### Interpretations of ^15^N‐SIP determinations in relation to complex N dynamics in soils

Due to the dynamic nature of the soil system any estimates of ^15^N in the soil protein pool represent the balance of assimilation into/loss from the pool at a given point in time. ^15^N incorporated into the soil protein pool does not simply accumulate with time, but is turned over as native soil N turns over, e.g. via catabolic mineralisation. Insights into the dynamics of this aspect of the N cycle in soil can now be gained at the AA level. In these experiments, applied labile substrates ([NH_4_]^+^ and Glu) are initially assimilated rapidly, with the amount assimilated increasing considerably between each time point until a transient equilibrium with slower soil N turnover/loss develops (Figs. 3(a), 3(d) and 4). For [NO_3_]^–^ (an energy‐demanding substrate), on the other hand, the dynamics are more complex and the rate of assimilation is always closer to that of turnover (Figs. 3(b) and 4). In natural systems these assimilation‐turnover dynamics would be subject to external forcings (e.g. rainfall events, soil type, etc.). Time‐course incubations of this type allow the overall assimilation‐turnover dynamics of the substrate with time, and other environmental variables, to be investigated and provide a measure of substrate availability/lability and value (via rate of incorporation and flux). Although this approach cannot currently generate absolute values for the assimilation of an applied ^15^N substrate by the soil microbial biomass or the amount of newly synthesized soil protein, it does provide enhanced insights compared to other currently available methods. It is reassuring that the percentages of applied ^15^N recovered in soil microbial biomass N studies (e.g.^11^, ^13^) are comparable (0.8–15.3% across these two studies) to those obtained herein.

## Conclusions

The novel compound‐specific ^15^N‐SIP approach using GC/C/IRMS described herein to investigate the fate of N amendments (e.g. via fertilisation; Fig. 1) in soils offers a number of advantages that existing techniques cannot to reveal a range of new insights, in particular:

- The method provides a sensitive and relatively selective means of assessing microbial assimilation of ^15^N‐labelled substrates/amendments applied at environmentally relevant concentrations and appropriately low ^15^N enrichments to minimise perturbations and ^15^N discrimination/isotopic fractionation, respectively. Bulk N isotope analysis cannot provide such insights and GC/MS ^15^N‐SIP studies require highly ^15^N‐labelled substrates for sufficient product AA ^15^N enrichment, making such studies more susceptible to ^15^N discrimination/isotopic fractionation effects and much more expensive.
- Valuable insights into microbial biochemical assimilation pathways can be gained and differences are readily revealed in the microbial processing of N‐containing amendments of differing chemical/biochemical natures, e.g. inorganic versus organic or different types of inorganic or organic amendment.
- Estimates are provided for newly synthesised soil protein, which are inaccessible based on currently available methods.
- Detailed quantitative insights can be gained into the dynamics of N cycling from an applied substrate through the soil protein pool.
- Scope exists for using this new approach to probe soil N cycling in relation to a wide range of soil biota, ecosystem variables and anthropogenic management regimes. Opportunities for further refinement of the method are exemplified by our recent paper,^43^ wherein additional insights were gained by considering different soil protein fractions.
- The method is potentially adaptable to investigate N cycling into other N‐containing biochemical pools, e.g. amino sugars.
